# Glassy carbon electrodes modified with multiwalled carbon nanotubes for the determination of ascorbic acid by square-wave voltammetry

**DOI:** 10.3762/bjnano.3.45

**Published:** 2012-05-10

**Authors:** Sushil Kumar, Victoria Vicente-Beckett

**Affiliations:** 1 Central Queensland University, Centre for Plant and Water Science, Rockhampton, Queensland 4702, Australia; 2 Central Queensland University, Centre for Environmental Management, Rockhampton, Queensland 4702, Australia

**Keywords:** ascorbic acid, carbon nanotubes, glassy carbon electrode, square-wave voltammetry

## Abstract

Multiwalled carbon nanotubes were used to modify the surface of a glassy carbon electrode to enhance its electroactivity. Nafion served to immobilise the carbon nanotubes on the electrode surface. The modified electrode was used to develop an analytical method for the analysis of ascorbic acid (AA) by square-wave voltammetry (SWV). The oxidation of ascorbic acid at the modified glassy carbon electrode showed a peak potential at 315 mV, about 80 mV lower than that observed at the bare (unmodified) electrode. The peak current was about threefold higher than the response at the bare electrode. Replicate measurements of peak currents showed good precision (3% rsd). Peak currents increased with increasing ascorbic acid concentration (dynamic range = 0.0047–5.0 mmol/L) and displayed good linearity (*R*^2^ = 0.994). The limit of detection was 1.4 μmol/L AA, while the limit of quantitation was 4.7 μmol/L AA. The modified electrode was applied to the determination of the amount of ascorbic acid in four brands of commercial orange-juice products. The measured content agreed well (96–104%) with the product label claim for all brands tested. Recovery tests on spiked samples of orange juice showed good recovery (99–104%). The reliability of the SWV method was validated by conducting parallel experiments based on high-performance liquid chromatography (HPLC) with absorbance detection. The observed mean AA contents of the commercial orange juice samples obtained by the two methods were compared statistically and were found to have no significant difference (*P* = 0.05).

## Introduction

L-ascorbic acid (AA), also known as vitamin C, is a well-known antioxidant, which helps the human body to reduce oxidative damage and protects food quality by preventing oxidative deterioration [1–3]. The overall oxidation of AA is [2]

[1]
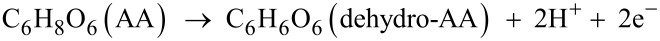


The growing use of AA in the food, pharmaceutical and cosmetic industries and its significance in biomedical science require the development of reliable, rapid and preferably portable analytical methods to quantify AA during the production and quality-control stages and in clinical applications [3–5]. Several methods for the determination of ascorbic acid concentration have been reported, such as HPLC [6], enzymatic analysis [7] and spectrophotometry [7]. However, these methods are relatively time-consuming and/or expensive.

The spectrophotometric method suffers from poor selectivity due to interference from other compounds present in commercial fruit juices (e.g., sugars or glucuronic acid) while citrate may affect enzymatic methods [7]. Electrochemical techniques, particularly cyclic voltammetry (CV) and square-wave voltammetry (SWV), have been employed as alternative tools for the evaluation of antioxidant activity [8]. These methods are attractive because of the speed of analysis, simplicity and low cost of the instrumental requirements.

Ascorbic acid oxidation at a bare glassy carbon electrode (GCE) generally occurs at a relatively high oxidation potential (e.g., 400 mV versus Ag/AgCl electrode), indicating a slow electron-transfer rate at the GCE [9]. Such sluggish electrode kinetics may also be due to electrode fouling caused by the deposition of oxidation product(s) of AA on the electrode surface, which results in poor selectivity and reproducibility, thus limiting the use of bare GCEs in quantitative measurements. Presently there are increasing reports on the use of carbon nanotubes (CNTs) in electroanalysis [10].

CNTs may be multiwalled or single-walled depending on the number of layers of carbon atoms in the nanotubes [11–12]. CNTs have unique geometric, mechanical, electronic and chemical properties. They possess a high aspect ratio (length/diameter) [13] and large surface areas (typically 200–300 m^2^/g) and, hence, potentially high electroactivity [14]. The defects present at the open ends of the CNTs have been observed to produce relatively low peak potentials and high peak currents in the voltammetry of several electroactive molecules at electrodes modified with CNTs [14–15]. Nafion, a perfluorosulfonated polymer with cation-exchange properties, has been used to stably confine insoluble particles on electrode surfaces as well as to protect the electrode from fouling during electrochemical studies, thus improving the performance of the modified electrode [16–18]. Multiwalled CNT (MWCNT)-modified GCEs exhibited signals enhanced by about five-fold in the detection of dopamine in the presence of AA [17]. Jacobs et al. [19] reviewed the use of MWCNTs to obtain enhanced signals in the detection of substances such as carbohydrates, nucleic acids, glucose, pesticides, and serotonin, with similar reports relating to trace metals [20] and nitroaromatic compounds [21].

This study reports the use of a MWCNT-modified GCE for the direct analysis of ascorbic acid by SWV and the application of the method to the analysis of ascorbic acid in commercial orange-juice products. The reliability of SWV method was validated against HPLC, an independent non-electrochemical analytical technique.

## Results and Discussion

### CV and SWV at a bare GCE

**Figure 1:**
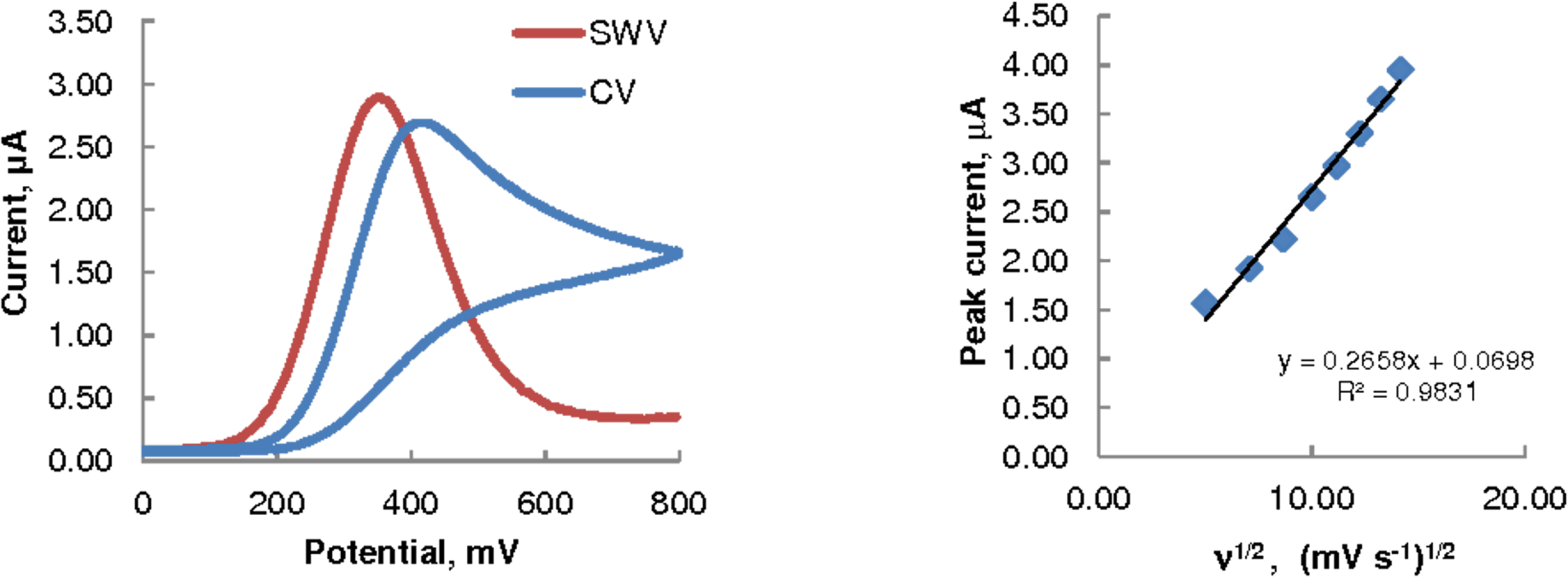
Typical CV and SWV voltammograms (left) at a bare GCE, 2 mmol/L AA in 0.1 M acetate buffer (pH 3.7), scan rate = 75 mV/s; dependence of CV peak currents on the scan rate ν (right).

CV provides an excellent and convenient tool to determine whether an electrochemical reaction is diffusion-controlled or kinetically controlled. The oxidation of 2 mmol/L ascorbic acid by using CV at a bare GCE was studied over the potential scan rates 25–200 mV/s. The left panel of Figure 1 shows a typical CV scan and a SWV scan for comparison. The plot on the right panel shows a good linear relationship between the observed CV peak current and the square root of the scan rate (ν^1/2^), demonstrating that the oxidation process is diffusion-controlled [22].

The mean peak potentials for the oxidation of ascorbic acid occurred at 418 mV (±7.2% rsd) and at 395 mV (±6.6% rsd) in CV and SWV, respectively. The net peak current was slightly higher (by ca. 7%) in SWV than in CV at all concentrations of ascorbic acid studied. The small negative shift (23 mV) of the peak potential observed in SWV relative to that observed in CV suggests that the applied square-wave potential helped maintain the electrode surface activity (through reduced adsorption of the oxidation products), resulting in a more favourable AA oxidation process. SWV was therefore chosen in this study for the development of the analytical method for AA analysis.

### GCE surface modification

**Figure 2:**
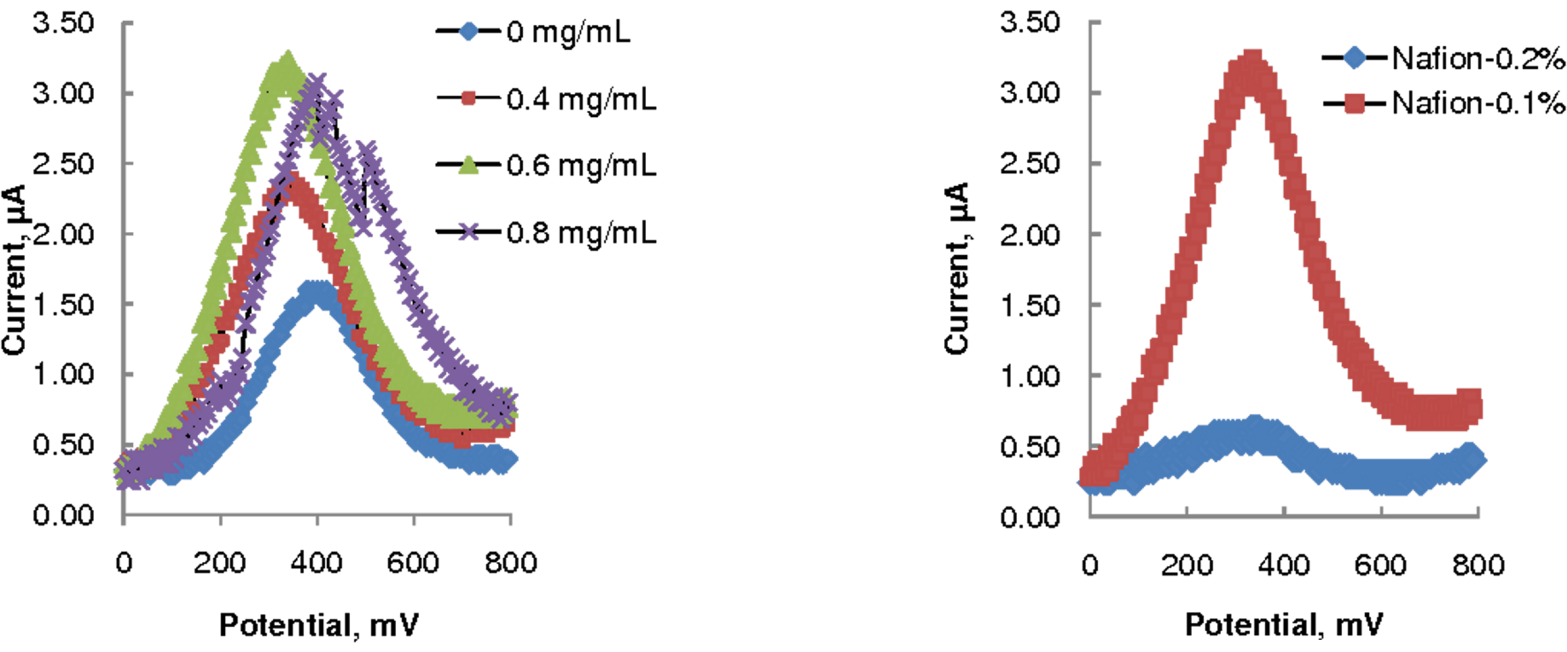
SWV of 1 mmol/L AA (in 0.1M acetate buffer, pH 3.7), scan rate = 75 mV/s: influence of varying the MWCNTs concentration (left) and Nafion concentration (right).

The effect of using an increasing concentration of MWCNTs in 0.1% (w/v) Nafion solution to modify the GCE is depicted in the voltammograms of 1 mmol/L AA in 0.1 M acetate buffer presented in Figure 2. The left panel shows that the maximum current response occurred at 0.6 mg/mL MWCNTs, which was nearly twice the response observed at the electrode modified only with Nafion (0.0 mg/mL MWCNTs). At 0.8 mg/mL MWCNTs the current was not stable, and no voltammogram could be recorded at 1 mg/mL MWCNTs. This suggests that at the higher concentrations the MWCNTs aggregated on the GCE could not be efficiently retained by the Nafion membrane, leading to a rather unstable layer structure [23–24]. Therefore, 0.6 mg/mL MWCNTs was employed in further SWV experiments.

With the MWCNT concentration fixed at 0.6 mg/mL, the influence of Nafion concentration (0.05–0.2%) on the voltammetric response was studied. No stable response could be recorded at 0.05% (w/v) Nafion. Stable responses were obtained at 0.1% (w/v) Nafion, but dramatically lower responses were recorded at 0.2% (w/v) Nafion. The lowest Nafion concentration was apparently unable to keep the MWCNTs attached to the GCE, whereas at the highest concentration the Nafion membrane was probably too thick with the result that it inhibited access of the analyte to the electrode. Further electroanalytical experiments therefore used GCE modified with 0.6 mg/mL MWCNTs in 0.1% (w/v) Nafion. The stability of the modified electrode was demonstrated in 100 CV scans (between 0 and 800 mV at 75 mV scan rate) in 0.25 mmol/L AA. The modified electrode showed only a 4% decrease in peak current over the 100 cycles, thus demonstrating a very stable and effective MWCNTs/Nafion film on the GCE.

### Effect of pH

**Figure 3:**
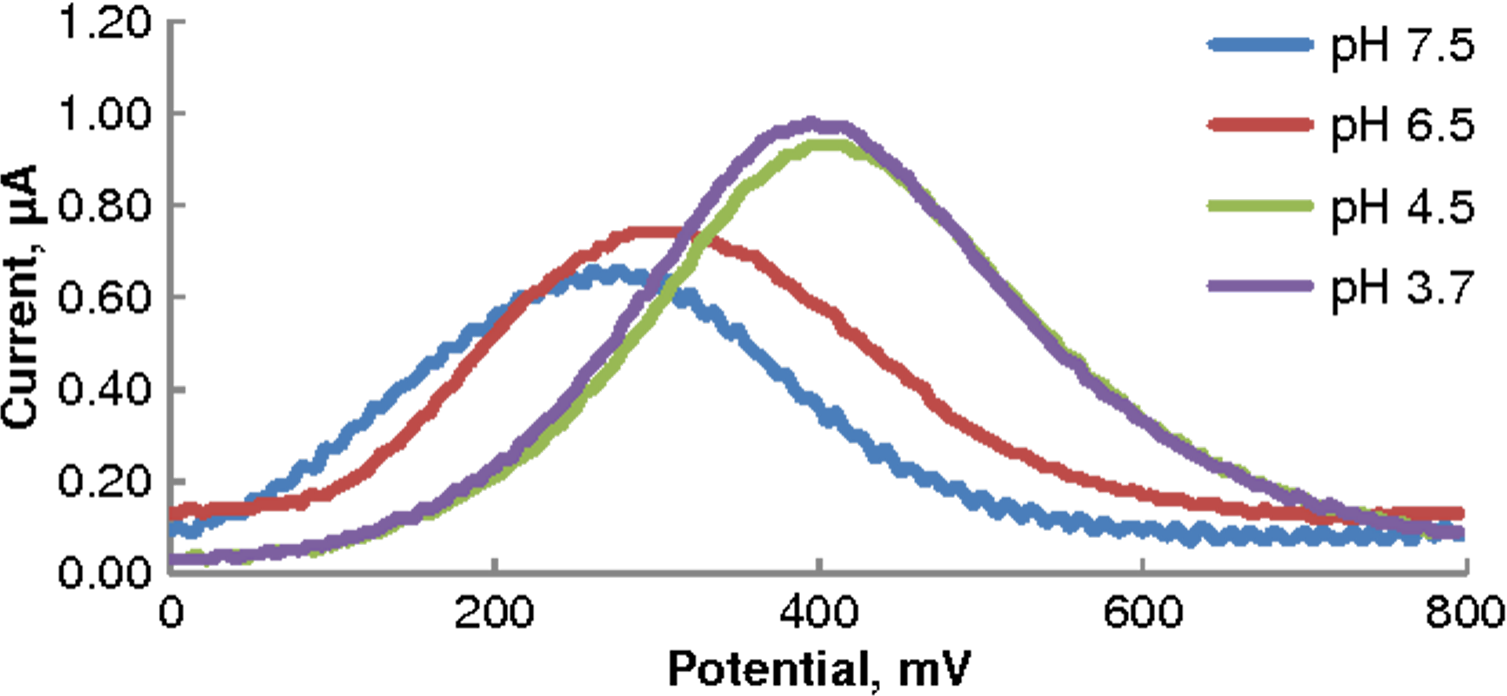
SWV on MWCNT–GCE, 0.25 mmol/L AA in different pH media (phosphate buffer, pH 6.5 and 7.5; acetate buffer, pH 3.7 and 4.5); scan rate = 75 mV/s.

The influence of pH on the oxidation of ascorbic acid was investigated over the pH range 3.7–7.5. The highest SWV response for AA oxidation was observed at pH 3.7; the peak currents were found to decrease with increasing pH (Figure 3). At pH 3.7 molecular ascorbic acid is present in a relatively large proportion (estimated to be about 72% at pH 3.7 for p*K*_a1_ = 4.10 and p*K*_a2_ = 11.79 [25]), making it compatible with the cation-exchange nature of the Nafion film; pH much higher than p*K*_a1_ would produce more anionic AA, which would be repelled by the Nafion membrane. The relatively low pH apparently also helped neutralise some of the negative charge on the Nafion surface. All subsequent SWV analyses were performed in 0.1 M acetate buffer (pH 3.7).

### Analytical performance of the SWV method

**Figure 4:**
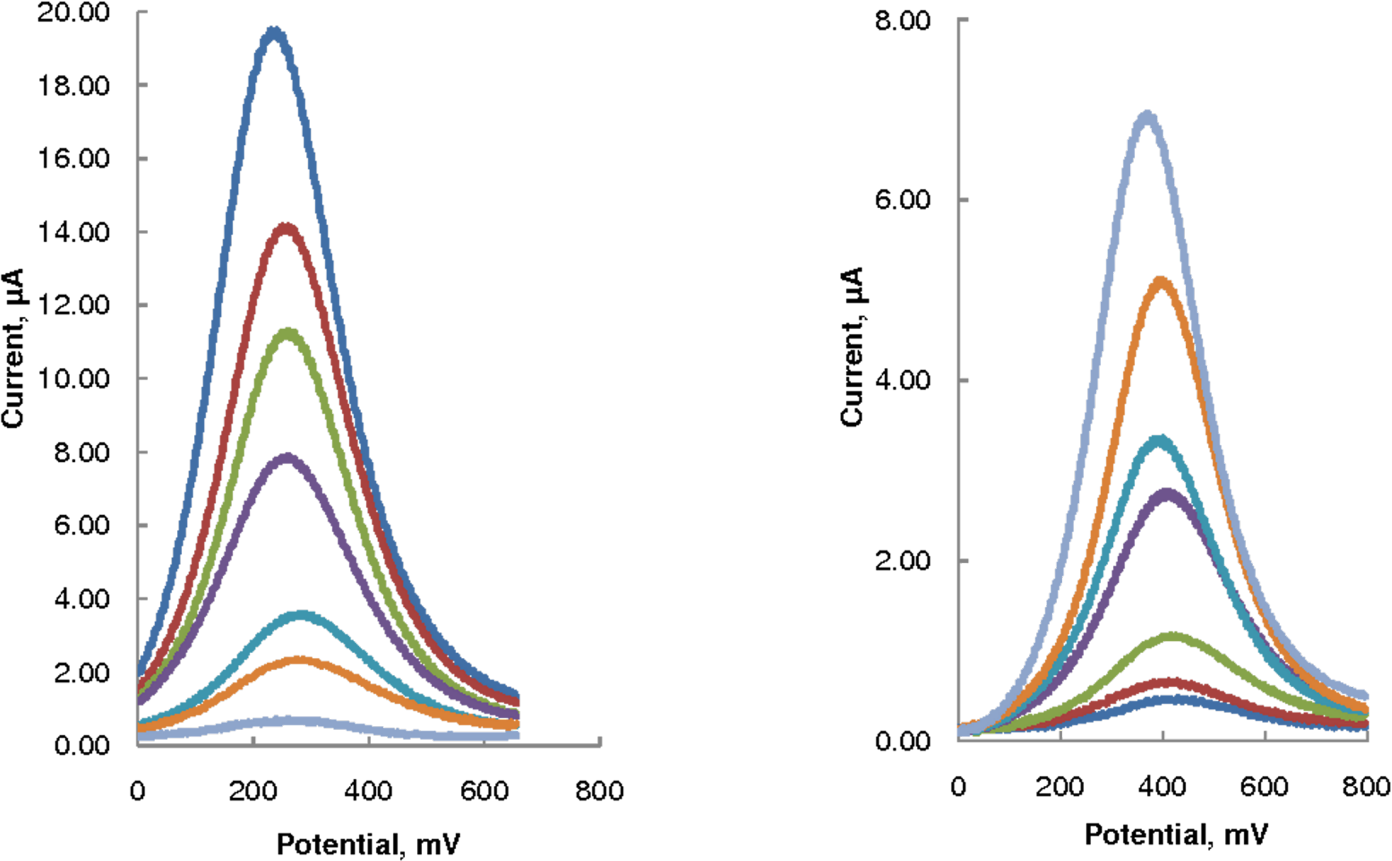
SWV in increasing AA concentrations (0.25–5.0 mmol/L in 0.1 M acetate buffer, pH 3.7); scan rate = 75 mV/s; at MWCNT–GCE electrode (left) and at bare GCE (right). (See Table 1 for the linear characteristics of the corresponding plots of the peak current vs AA concentration.)

Figure 4 shows SWV voltammograms in 0.25–5.0 mmol/L AA, with peak currents increasing linearly with AA concentration. The MWCNT–GCE responses were nearly three-fold higher than those obtained at the bare GCE, demonstrating the electrocatalytic action of MWCNTs. The peak potentials at the MWCNT–GCE shifted negatively by almost 80 mV compared with those obtained on the bare GCE, consistent with the observation of Fei et al. [15], who used CV at a GCE modified with a composite film of single-walled carbon nanotubes and dihexadecyl hydrogen phosphate for the determination of ascorbic acid concentration and reported a negative shift of up to 468 mV.

Table 1 shows that the calibration plot (dynamic concentration range = 0.0047–5.0 mmol/L) obtained on MWCNT–GCE showed better linearity (*R*^2^ = 0.994) than that on the bare GCE (*R*^2^ = 0.984). In addition, the calibration sensitivity (the slope of the regression equation) for the modified electrode was 2.8 times higher than that of the bare electrode. Table 1 also shows that better repeatability and reproducibility of both peak currents and peak potentials were observed on the modified GCE compared to the bare GCE, demonstrating clearly that the modified electrode was protected from fouling by the Nafion membrane.

**Table 1:** Analytical performance of the SWV method.

analytical parameter	MWCNT–GCE	bare GCE

**dynamic AA concentration range** (mmol/L)	0.0047–5.0	0.028–5.0
**calibration sensitivity** (slope of regression equation)	3.71	1.32
***R*^2^**	0.994	0.984
**LOD** (µmol/L)	1.4	8.3
**LOQ** (µmol/L)	4.7	28
**mean peak potential** (*n* = 5) and repeatability (% rsd), 1 mmol/L AA	313 mV (±3.8%)	395 mV (±6.6%)
**mean peak current** (*n* = 5) and repeatability (% rsd), 1 mmol/L AA	3.41 µA (±3.5%)	1.17 µA (±7.5%)
**mean peak potential** (*n* = 5) and reproducibility (% rsd), 1 mmol/L AA	313 mV (±3.4%)	387 mV (±6.8%)
**mean peak current** (*n* = 5) and reproducibility (% rsd), 1 mmol/L AA	3.13 µA (±3.2%)	1.21 µA (±6.3%)

The limit of detection (LOD) (based on 3 × standard deviation of the blank) and the limits of quantitation (LOQ) (based on 10 × standard deviation) were much lower for the modified electrode than for the bare GCE (Table 1). The estimated LOD was better than that (2.8 μmol/L) reported by Zhang and Jiang [26], who used CV at a glassy carbon electrode modified with gold nanoparticles for the analysis of AA.

### Analysis of ascorbic acid in commercial orange-juice products

#### Calibration-curve technique

**Figure 5:**
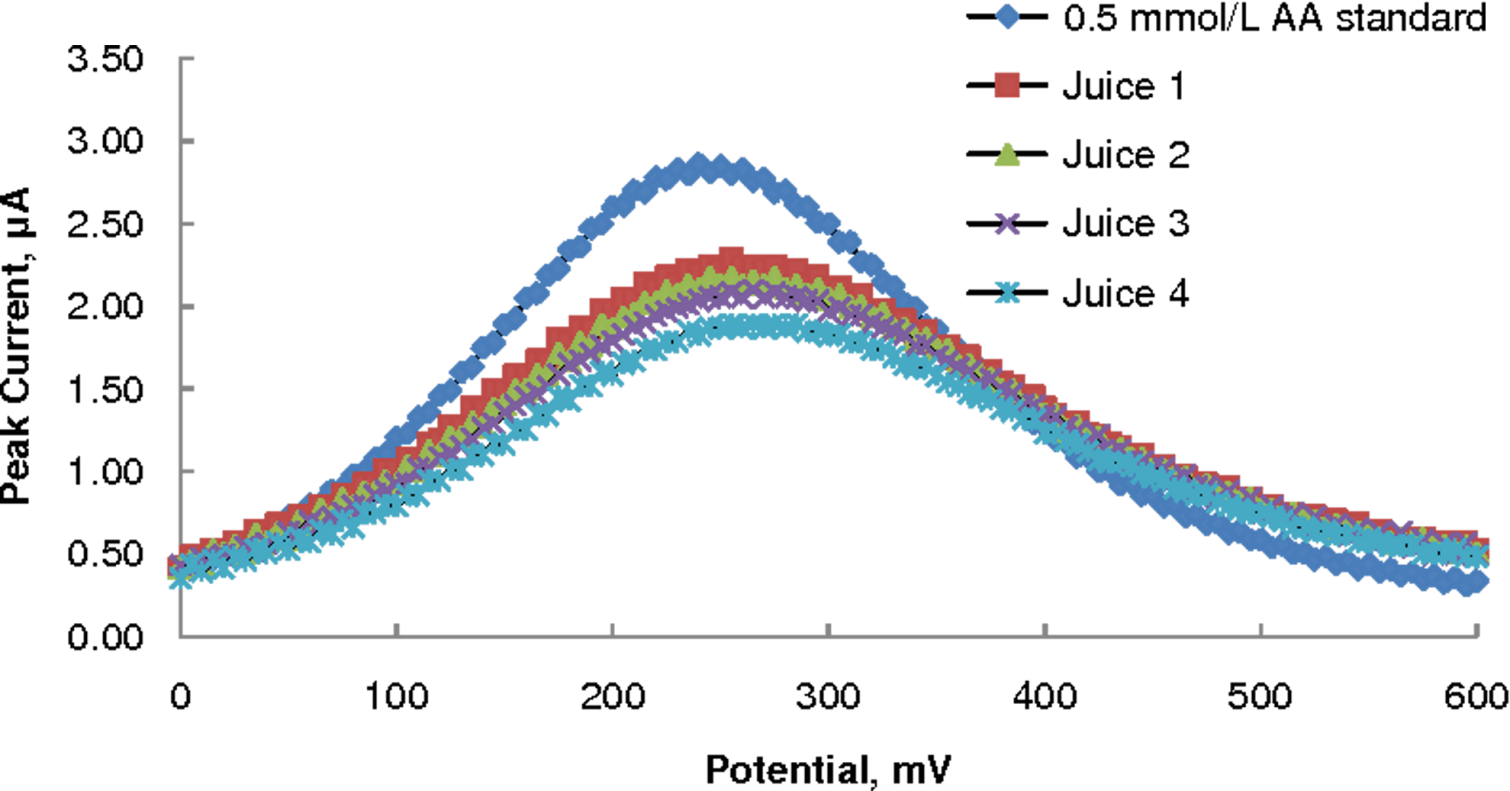
SWV voltammograms of four commercial orange juice samples (diluted by 1/5) in acetate buffer, pH 3.7; 0.5 mmol/L AA standard shown for comparison.

The ascorbic acid contents of four brands (labelled simply as 1–4) of commercial orange-juice products were determined by SWV on MWCNT–GCE using the external-standard calibration technique. Typical SWV voltammograms are shown in Figure 5 and the results are presented in Table 2 (together with those obtained by the HPLC method, which are discussed later). It is seen that the measured AA content of the (1/5) diluted juice samples agreed very well (96–104%) with the claim on the product label.

**Table 2:** Ascorbic acid content of commercial orange-juice samples.

	juice 1	juice 2	juice 3	juice 4

**AA concentration** (1/5 product-label claim)	0.454 mmol/L (8 mg/100mL)			
**mmol/L AA** (1/5)-diluted sample, SWV ± sd	0.435 ± 0.011	0.474 ± 0.016	0.451 ± 0.010	0.469 ± 0.017
**% AA** (relative to label claim), SWV	95.8	104.4	99.3	103.3
**mmol/L AA** (1/5)-diluted sample, HPLC ± sd	0.462 ± 0.011	0.439 ± 0.009	0.464 ± 0.015	0.461 ± 0.011
**% AA** (relative to label claim), HPLC	101.8	96.7	102.2	101.5
**mmol/L AA** spiked sample (SWV)	0.951	0.973	0.912	0.981
**% recovery** (SWV)	103	99.8	92.2	102.4
**mmol/L AA** spiked sample (HPLC)	0.965	0.931	0.942	0.951
**% recovery** (HPLC)	101	98.4	95.6	98.0

#### Recovery test

Recovery tests were performed to establish the reliability of the SWV method. The diluted juice samples were spiked with 0.5 mmol AA and analysed. It is seen from Table 2 that, with the exception of juice 3, excellent recoveries of the spiked amount (92–103%) were obtained by SWV.

#### SWV using the standard-addition technique

**Figure 6:**
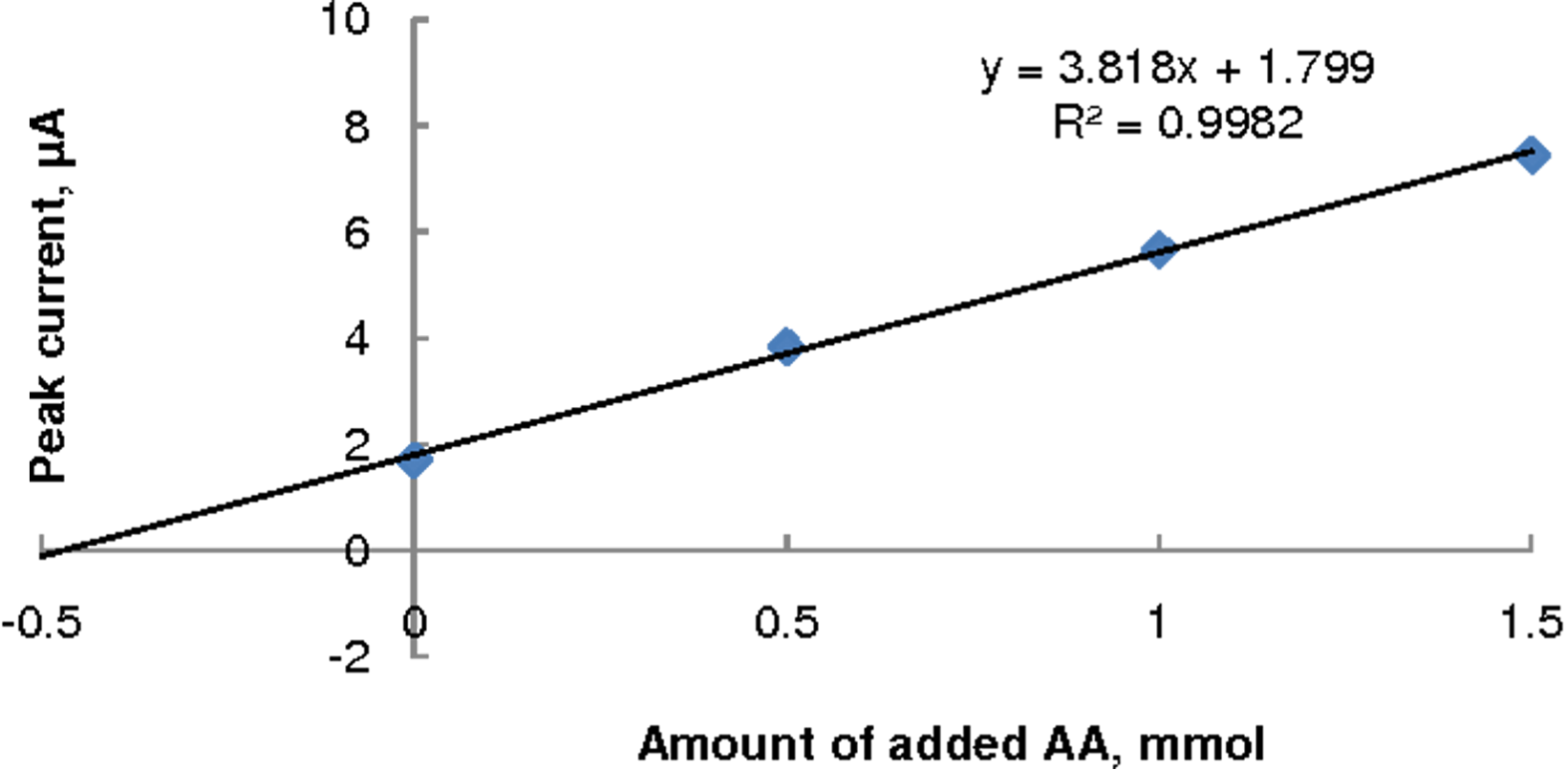
Standard addition plot of SWV data for (1/5)-diluted juice 3.

The standard-addition technique (Figure 6) was applied in the analysis of the juice 3 (which gave the lowest recovery) to verify whether there were any matrix effects. The AA concentration of the (1/5)-diluted juice was found to be 0.471 mmol/L. Since this differed only by 4.4% from the result obtained by the calibration-curve technique (Table 2) no significant matrix effects were evident in the analysis.

#### HPLC of ascorbic acid

**Figure 7:**
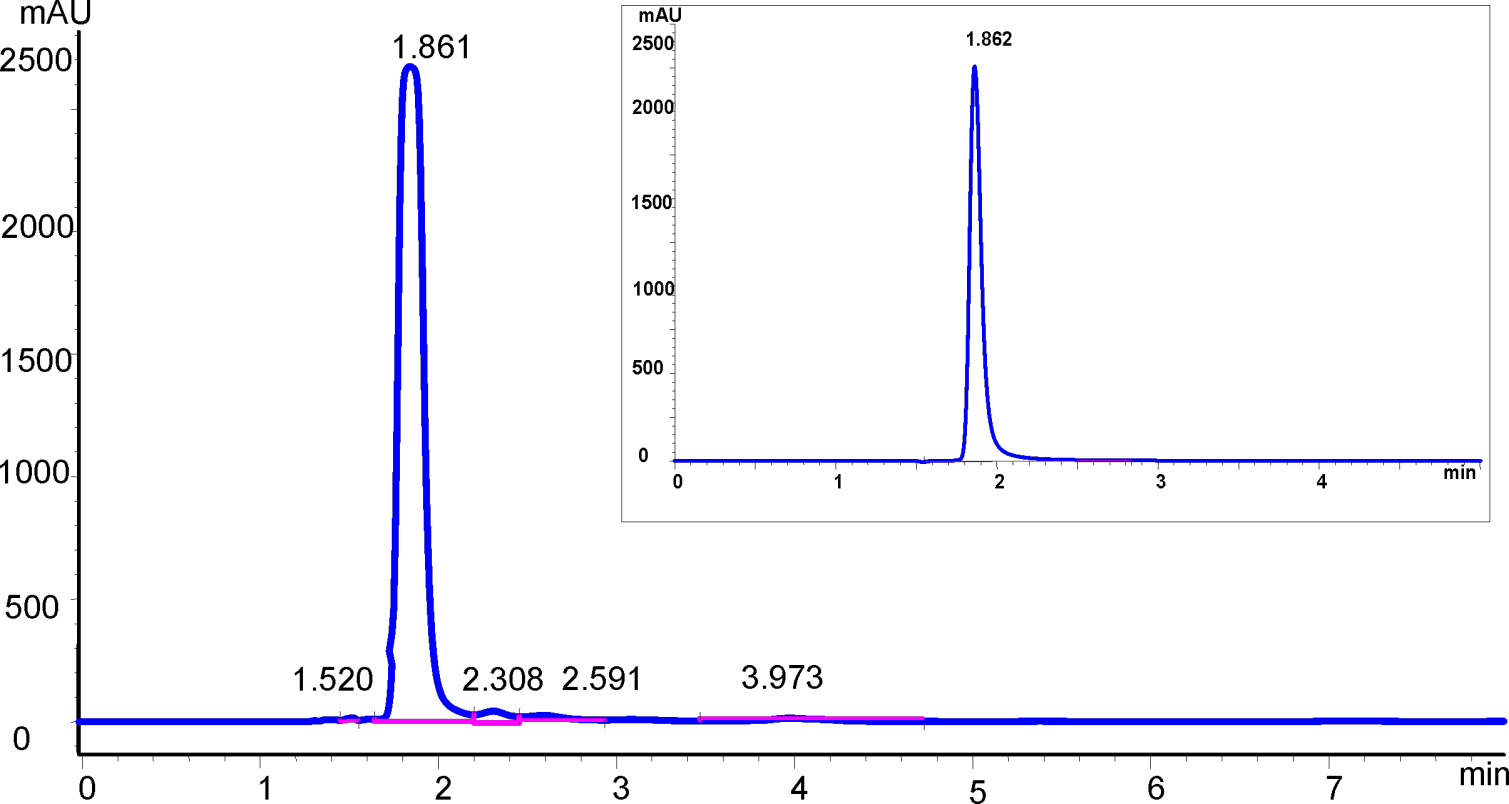
Typical HPLC chromatograms of sample of (1/5)-diluted juice 1 and 0.5 mmol/L AA standard (inset).

Typical chromatograms of a juice sample and the AA standard (retention time = 1.862 min) are shown in Figure 7. An excellent linear correlation (*R*^2^ = 0.998) between the AA peak area and standard AA concentrations (dynamic concentration range = 0.0023–5.0 mmol/L) was observed. The method showed excellent precision (2.1% and 2.4% for repeatability and reproducibility, respectively). The better precision of the HPLC data compared to the SWV data is attributed to the auto-injection facility of the HPLC equipment. The LOD and LOQ were 0.7 and 2.3 µmol/L, respectively. The LOD is approximately half that (1.53 µmol/L) reported by Burini [27], who used a C_18_ column with a mobile phase of 80 mM phosphate buffer (pH 7.8) and methanol.

Triplicate HPLC analyses of the same four brands of commercial orange juice were performed. The chromatograms showed AA eluting at 1.86 min with no major additional peaks observed (Figure 7). The percentage recoveries in the spiked samples (see Table 2) analysed by HPLC were excellent (95–101%). Fresh fruit juices may contain a number of organic acids (e.g., citric acid) and sugars (e.g., glucose, fructose and sucrose), which could cause potential interferences. However no such interferences were evident in the analyses.

#### Comparison of the SWV method with the HPLC method

The results of the HPLC method were compared statistically with those of the SWV method. Table 3 shows that the calculated *F*-value for each juice analysed was less than the tabulated *F-*value (*P* = 0.05) for a two-tailed test, indicating that the precisions of the means of the two methods (SWV and HPLC) were not significantly different for all of the juice samples studied. Comparison of the mean (three replicates) AA content of each commercial orange juice obtained by the two methods (SWV and HPLC) using the *t*-test showed that for each juice analysed there was no significant difference (*P* = 0.05) between the mean values obtained by the two methods (see Table 3).

**Table 3:** Comparison of the results of SWV and HPLC methods.

statistic	juice 1	juice 2	juice 2	juice 4

**calculated *F***	1.15	3.42	2.13	2.32
**tabulated *F*** (*P* = 0.05, 2-tailed) [28]	39			
**calculated *t***	2.01	2.24	1.22	1.83
**critical *t*** (*P* = 0.05) [28]	4.303			

## Conclusion

The MWCNTs-modified GCE was stable over 100 CV runs with the peak currents decreasing by only 4%. The SWV method at the bare GCE was found to be more sensitive than CV for the determination of the concentration of ascorbic acid. The excitation by a square-wave potential probably helped minimise fouling of the electrode surface caused by the oxidation products of AA, which probably caused the poorer reproducibility and sensitivity observed in the CV technique. The SWV method on MWCNT–GCE for AA analysis showed good analytical performance in terms of linearity, repeatability and reproducibility, and limit of detection or quantitation. The AA oxidation peak potential observed in SWV shifted negatively by 80 mV at the modified GCE compared with bare GCE, indicating a more favourable oxidation process in the presence of MWCNTs. The sensitivity of the modified GC electrode increased by a factor of 2.8 and is attributed to the increased surface area provided by the MWCNT coating on the GCE, and to enhanced electrochemical activity from edge-plane defects at the MWCNTs facilitating the electron transfer. The LOD and LOQ of the SWV method at the MWCNT–GCE were only twice the limits obtained with the HPLC method. This is rather impressive considering the much simpler SWV instrumentation compared to the HPLC system. The SWV method proved its reliability in the analyses of AA in samples of orange juice, with good recoveries comparable to those obtained by HPLC. The mean AA contents obtained by the two methods were not significantly different (*P* = 0.05). The SWV method has clear advantages over the HPLC method: it is simple, precise, reliable and rapid, and notably (for the application tested) did not require any special pretreatment of the sample. The analysis time spent on SWV manipulations and measurement was less than 15 min, making the method suitable for routine analyses. Using CNTs to modify conventional electrodes (such as the popular GCE) is a simple and effective approach to enhance the electrode sensitivity for trace analyses.

## Experimental

### Electrodes and electrochemical instrumentation

All voltammetric measurements were performed in a 20 mL glass vial with a lid that had ports to accommodate the three mini electrodes (Cypress System, Chelmsford, MA, USA): the GCE (*d* = 1 mm) working electrode, platinum-wire auxiliary electrode (*d* = 0.5 mm), and a Ag|AgCl (leak-free) reference electrode. All potentials are reported against the Ag|AgCl reference electrode. Cyclic and square-wave voltammetric experiments were carried out at room temperature (23–25 °C) by using a MacLab potentiostat interfaced to a PowerLab 400 and controlled by the EChem v1.5 software (all components from eDAQ, Denistone, NSW, Australia). SWV (SW amplitude = 15 mV, frequency = 20 Hz) voltammograms of ascorbic acid were recorded from 0–800 mV at a scan rate of 75 mV/s.

### Reagents

The multiwalled carbon nanotubes (diameter: 6–13 nm, length: 2.5–20 μm, purity > 99.8%) and Nafion (5% (w/v) in a mixture of lower aliphatic alcohols) were purchased from Sigma Aldrich (St. Louis, MO, USA). L-ascorbic acid was obtained from BDH Chemicals (Port Fairy, VIC, Australia). All other chemicals (HCOOH, K_2_HPO_4_, KH_2_PO_4_, KOH, CH_3_COOH, C_2_H_3_NaO_2,_ and HNO_3_) used in this work were of analytical reagent grade and obtained from Ajax Chemicals (Sydney, NSW, Australia). All standard solutions were prepared with ultrapure Milli-Q water (18.2 MΩ·cm; Milli-Q System, Millipore, Molsheim, France).

### Electrode Preparation

Prior to its use or modification with MWCNTs, the working GCE was polished on a microcloth (Buehler**,** Lake Bluff, IL, USA) in a slurry of 0.05 µm alumina (Buehler, Lake Bluff, IL, USA) in Milli-Q water until the surface showed a mirror-like finish. The electrode was then rinsed with Milli-Q water, sonicated for 1 min to remove trace alumina particles from the electrode surface, and then air dried. This cleaning procedure was applied before all voltammetric measurements were carried out. The platinum-wire auxiliary electrode was typically polished with a CarbiMet fine-grit polishing disc (Buehler, Lake Bluff, IL, USA) to remove any oxides of platinum formed on its surface, immersed in 10% (v/v) nitric acid for about 30 s, rinsed thoroughly with Milli-Q water and then air dried. The reference electrode was cleaned by thoroughly rinsing the tip with Milli-Q water and then air dried.

To determine the optimum concentration of MWCNTs needed to modify the glassy carbon electrode, suspensions of 0.0, 0.4, 0.6 and 1.0 mg MWCNTs were dispersed in separate 1 mL aliquots of 0.1% (w/v) Nafion/ethanol and sonicated for 1.5 h. On the polished GCE, 5 µL of each suspension were applied evenly, and the ethanol was allowed to evaporate at room temperature for 1 h. The modified electrode was then washed repeatedly with Milli-Q water to remove any remaining modifying solution and kept at room temperature until use. The influence of Nafion concentration was determined by varying its concentration (0.05, 0.10, 0.20% (w/v) in ethanol) while keeping the MWCNTs at 0.6 mg/mL. The homogeneity of the dispersion of MWCNTs in the Nafion film (at the optimum concentrations of MWCNTs and Nafion) was ascertained by applying a few microlitres of the modifying solution on a glass slide, allowing the ethanol to evaporate and viewing the dried film through a digital optical microscope.

### HPLC analyses

HPLC analyses were performed on an Agilent 1200 system (Agilent Technologies Pty Ltd Australia, Mulgrave, VIC) controlled by the Agilent ChemStation software. The column was an Agilent Zorbax Eclipse XDB-C_18_ (15 × 0.46 cm, particle size = 5 μm, total carbon content = 10%, surface area = 1800 m^2^/g and average pore diameter = 80 nm). The chromatographic conditions used were: mobile phase 0.1% (v/v) formic acid in Milli-Q water [29], flow rate 1 mL/min, injection volume 20 µL and detection wavelength = 245 nm.

### Standard AA solutions; repeatability or reproducibility tests

Stock (250 mmol/L) standard AA solutions were prepared either in 0.1 M phosphate buffer (pH 6.5 or pH 7.5) or in 0.1 M acetate buffer (pH 3.7 or pH 4.5). Five replicate SWV runs in 1 mmol/L AA were conducted on a single day (to test the repeatability of the SWV response) and over five days, by using freshly prepared modified electrodes and AA solutions (to assess reproducibility). For HPLC work, stock standard (250 mmol/L) AA solution was prepared in 0.1% (v/v) formic acid/Milli-Q water (pH 2.7 approx). Three samples of freshly prepared 0.5 mmol/L AA solutions were run on one day to establish the repeatability of the analytical response, while similar fresh preparations were run over three different days to establish reproducibility of the analytical response.

### Analysis of commercial orange-juice products

Four brands (identified simply as 1–4) of commercial orange-juice products were obtained from a local supermarket. Juices were filtered through Whatman No.4 paper to remove fibre and pulp. For SWV, all juice samples were diluted (1/5) with 0.1 M acetate buffer (pH 3.7) for analysis. The same four brands of commercial orange juice were employed for HPLC analysis. The juice samples were likewise filtered to remove fibre and pulp. All juice samples were diluted (1/5) with 0.1% (v/v) formic acid/Milli-Q water for HPLC analysis.

### Recovery tests

Recovery tests were performed in triplicate. To aliquots of the orange-juice products representing (1/5)-diluted orange-juice samples, 2 mL of 250 mmol/L standard ascorbic acid (equivalent to a spiked amount of 0.5 mmol AA) were added and then diluted in 100 mL standard flasks with 0.1 M acetate buffer (pH 3.7) or with 0.1% (v/v) formic acid/Milli-Q water for analysis by SWV or HPLC, respectively.

### SWV by the standard addition method

Four lots of 100 mL solutions of (1/5)-diluted juice (i.e., containing 0.454 mmol/L of AA, as per the product-label claim) were prepared in 100 mL standard flasks. Aliquots of 0.0, 2.0, 4.0, 6.0 mL of 250 mmol/L standard AA solution were added into separate juice sample flasks representing 0.0, 0.5, 1.0 and 1.5 mmol of added AA and then diluted to volume.
